# Long-term dynamics of the human oral microbiome during clinical disease progression

**DOI:** 10.1186/s12915-021-01169-z

**Published:** 2021-11-06

**Authors:** Ana Duran-Pinedo, Jose Solbiati, Flavia Teles, Ricardo Teles, Yanping Zang, Jorge Frias-Lopez

**Affiliations:** 1 Department of Oral Biology, University of Florida, College of Dentistry, 1395 Center Drive, Gainesville, FL 32610-0424 USA; 2 Department of Basic & Translational Sciences, University of Pennsylvania, School of Dental Medicine, 240 South 40th Street, Philadelphia, PA 19104-6030 USA; 3 Department of Periodontics, University of Pennsylvania, School of Dental Medicine, 240 South 40th Street, Philadelphia, PA 19104-6030 USA; 4 Gene Expression & Genotyping Core, Interdisciplinary Center for Biotechnology Research, University of Florida, 178 B CGRC, 2033 Mowry Road, Gainesville, FL 32610 USA

## Abstract

**Background:**

Oral microbiome dysbiosis is linked to overt inflammation of tooth-supporting tissues, leading to periodontitis, an oral condition that can cause tooth and bone loss. Microbiome dysbiosis has been described as a disruption in the symbiotic microbiota composition’s stability that could adversely affect the host’s health status. However, the precise microbiome dynamics that lead to dysbiosis and the progression of the disease are largely unknown. The objective of our study was to investigate the long-term dynamics of periodontitis progression and its connection to dysbiosis.

**Results:**

We studied three different teeth groups: sites that showed disease progression, sites that remained stable during the study, and sites that exhibited a cyclic deepening followed by spontaneous recovery. Time-series analysis revealed that communities followed a characteristic succession of bacteria clusters. Stable and fluctuating sites showed high asynchrony in the communities (i.e., different species responding dissimilarly through time) and a reordering of the communities where directional changes dominated (i.e., sample distance increases over time) in the stable sites but not in the fluctuating sites.

Progressing sites exhibited low asynchrony and convergence (i.e., samples distance decreases over time). Moreover, new species were more likely to be recruited in stable samples if a close relative was not recruited previously. In contrast, progressing and fluctuating sites followed a neutral recruitment model, indicating that competition between closely related species is a significant component of species-species interactions in stable samples. Finally, periodontal treatment did not select similar communities but stabilized α-diversity, centered the abundance of different clusters to the mean, and increased community rearrangement.

**Conclusions:**

Here, we show that ecological principles can define dysbiosis and explain the evolution and outcomes of specific microbial communities of the oral microbiome in periodontitis progression. All sites showed an ecological succession in community composition. Stable sites were characterized by high asynchrony, a reordering of the communities where directional changes dominated, and new species were more likely to be recruited if a close relative was not recruited previously. Progressing sites were characterized by low asynchrony, community convergence, and a neutral model of recruitment. Finally, fluctuating sites were characterized by high asynchrony, community convergence, and a neutral recruitment model.

**Supplementary Information:**

The online version contains supplementary material available at 10.1186/s12915-021-01169-z.

## Background

The oral cavity harbors a large and complex community of beneficial microbes that remain stable over long periods [1]. As in other microbiomes, this complex assembly of organisms’ stability is critical for good health but remains almost entirely unexplored.

Periodontitis, one of the most common oral diseases globally, is an example of dysbiosis-driven disease, which results in an uncontrolled inflammation of the periodontal tissues, which can lead to tooth and bone loss [2]. Despite being studied for decades, our understanding of the ecological shift toward periodontitis initiation and progression is still limited.

That is due, in part, to the cross-sectional nature of previous clinical studies. Even though they have revealed compositional and functional dysbiosis of the oral microbiome in periodontitis [2–4], only longitudinal observations can shed light on the microbiome dynamics during disease progression. Understanding the oral microbiome’s temporal dynamics is integral in leveraging these microbial communities to promote human health.

Most microbiome stability studies have focused on the gut microbiome and used summary community metrics of diversity and distance to quantify and relate communities over time [5, 6]. However, these kinds of studies do not address the fundamental problem of what makes a microbiome stable. Dysbiosis is believed to be a critical factor in the onset of several microbiome-driven diseases [7–9]. Nonetheless, the study of microbial taxonomic profiles has limits in explaining dysbiosis, as these profiles can be highly divergent among patients, making it difficult to implicate specific microbial species or strains in disease onset and progression. To manipulate the microbiome to improve health, we need to understand community structure and composition, and we need models to quantify and predict the microbial community’s stability.

The mere definition of dysbiosis is problematic. It is such a broad concept that it could mean almost any change in microbiome compositions [10], and as Olesen and Alm indicate [11]: “the fact that healthy and ill people have different microbiomes is no longer a novel or useful observation. We need to show that differences in the microbiota can be used to predict or ameliorate disease, and not just show that differences exist.” Therefore, there is a need to identify overarching principles and patterns of microbiome behavior linked to the community’s stability.

To begin addressing this knowledge gap, we focused our interest on understanding the dynamics of long-term changes in the oral microbiome’s community structure rather than explaining the disease’s progression by changes in the relative abundance of specific organisms. Specifically, we hypothesized that changes in the microbiome members’ interconnections and their dynamics could be a complementary indicator of the disease’s outcome. Here, we study the oral microbiome’s long-term dynamics by systematically monitoring and sampling a cohort of the same 15 individuals presenting periodontitis during 1 year. In addition, specific teeth in the entire mouth of the patients enrolled in the study were sampled individually every 2 months. After the study was finalized, samples were classified according to their clinical profiles as stable if no changes in clinical measurements were observed during the study or progressing if clinical measurements indicated an exacerbation of the disease.

Additionally, we studied teeth that exhibited cyclic deterioration of clinical measurements followed by spontaneous recovery, a fluctuating behavior commonly observed in the clinic [12, 13]. The work described here characterized oral microbial stability by looking at changes in ecological elements of community organization. Temporal correlation network analysis revealed a higher degree of centrality and lower betweenness centrality in stable samples during the whole study.

We were able to identify the synchrony of the progressing samples as a defining element for these sites. Asynchrony among species can result in community stability if a rise in one compensates for a decline in another species. Therefore, the degree of community synchrony is an essential indicator of ecosystem properties’ stability [14]. Besides, we found that new species were more likely to be recruited in stable samples if a close relative was not recruited previously.

In contrast, progressing and fluctuating sites followed a neutral recruitment model, indicating that competition between closely related species is a significant component of species-species interactions in stable samples. Thus, in the progressing and fluctuating samples, cooperation could be driven by these interactions. Together, our findings show that the study of the oral microbiome dynamics in disease progression through time-series analysis can define overarching ecological principles that could better explain the evolution and outcomes of specific microbial communities in the oral cavity.

## Results

### Patterns of periodontitis progression across clinical groups

The data presented here were obtained from a prospective multi-center clinical study to identify periodontitis biomarkers (i.e., gum disease) progression described elsewhere [13, 15]. A total of 415 participants were examined every 2 months for 12 months in the absence of periodontal treatment to Additional File 5, Table S1monitor periodontal disease progression, based on clinical attachment level (CAL) measurements. Then, participants received periodontal therapy (scaling and root planning, SRP) and were followed for six more months. At each visit (baseline, 2, 4, 6, 8, 10, and 12 months, and 6 months post-treatment visits), participants received clinical examination and provided subgingival (i.e., below the gumline) microbial samples from the same specific teeth. At the end of the study, three clinical behaviors were observed on the teeth examined: stability, disease progression, and fluctuation (cycles of disease progression/regression) [13, 15].

Our approach is detailed in Additional File 1, Fig. S1a. Fifteen individuals were selected for the present study. Three teeth were chosen in each of them, each representing one of the three clinical groups (3 teeth/participant, 45 teeth total). For each tooth, we analyzed eight samples, representing each of the time points in the longitudinal study (baseline, 2, 4, 6, 8, 10, and 12 months, and 6 months post-treatment), for a total of 360 microbial samples included in this study. Complete details of the study outline are presented elsewhere [13, 15]. To assess whether a sample size of 15 subjects per group likely affords adequate statistical power, we calculated effect size measured as omega-squared (*ω*^2^) described in Kelly et al. [16] using Jaccard distance. This method has been specifically designed to estimate sample size for microbiome analysis. We found that with a power of 90%, the stable group has a *ω*^2^ of 0.019, the progressing group 0.042, and the fluctuating group 0.022; all smaller than the *ω*^2^ of 0.08 that Kelly et al. found in the ten subjects per group.

Time-series modeling and forecasting confirmed the validity of the three clinical trajectories selected to study. We employed the Dickey-Fuller test to determine whether the time series were stationary or non-stationary, using CAL in our predicted trajectories: stable (no change in CAL), progressing (an increase of CAL with time), and fluctuating (up and down changes in CAL), illustrated in Additional File 1, Fig. S1b. Both progressing and fluctuating were non-stationary. Autoregressive Integrated Moving Average (ARIMA) modeling [17], a widely used approach to stationary time-series forecasting, was used for stable samples, whereas differencing [17] was employed for progressing and fluctuating samples. We performed forecasting on CAL profiles as a proxy for disease progression with time for the ΔCAL in stable, progressing, and fluctuating samples (Additional File 2, Fig. S2). Forecasting results indicate that, without intervention, the three patterns previously defined in our samples followed the predicted classification (Additional File 2, Fig. S2b,d,f). The forecasted values for the next 12 months flatten in the stable samples (Additional File 2, Fig. S2b), the ΔCAL (difference of CAL values between time points) grows exponentially in the case of the progressing sites (Additional File 2, Fig. S2d), and it follows a zig-zagging trajectory in the fluctuating sites (Additional File 2, Fig. S2f). As expected, after periodontal treatment, CAL values were reduced in all groups (Additional File 2, Fig. S2a,c,e).

### Patterns of community structure during periodontitis progression across clinical groups

**Fig. 1:**
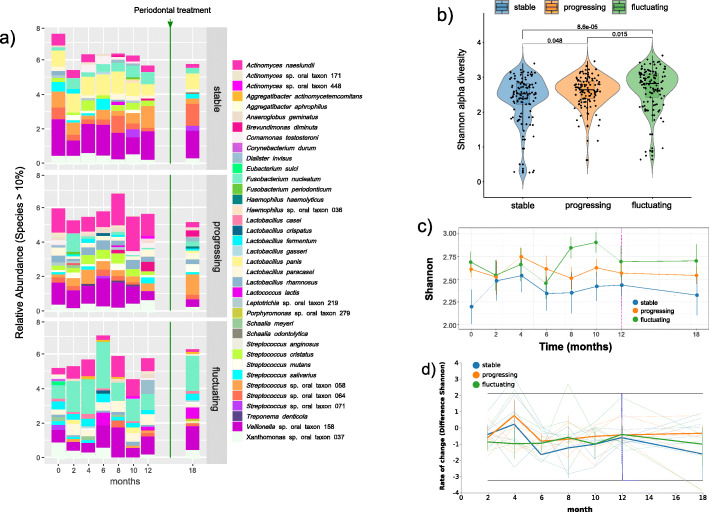
Taxonomy and diversity profiling of the different clinical groups of teeth according to disease progression. **a** Temporal changes in the relative frequency of the most abundant common taxa (> 10%) among all groups at the species level. Counts were edgeR normalized. The vertical green line indicates the moment when patients received periodontal treatment. **b** Violin plot of Shannon α-diversity of all samples in the three different groups. Boxes span the first to third quartiles; the horizontal line inside the boxes represents the median, and black dots represent all samples in each group. Pairwise comparison is performed using the non-parametric Wilcoxon test, and *p* values are displayed. **c** Profiles of Shannon diversity across time. Error bars represent standard deviation. The vertical blue line indicates the moment when patients received periodontal treatment. **d** Rate of change of Shannon index over time. We calculated the first differences, which is the magnitude of change between successive time points, and represented them on a volatility plot. Error bars represent standard deviation

We profiled prokaryotic composition for the 16S rRNA gene datasets and eukaryotic composition for ITS1 and ITS2 genes using Kraken2 and Bracken programs with a custom 16S rRNA+ITS database [18, 19]. The profiles of community composition in the three kinds of samples showed a similar composition of the most abundant taxa, although some species’ relative abundance was different (Fig. 1a). For instance, *Fusobacterium nucleatum* was more abundant in the fluctuating samples than in stable and progressing samples, whereas *Streptococcus* sp. oral taxon 064 and oral taxon 058 were more abundant in the stable and progressing samples than in the fluctuating (Fig. 1a). Progressing and stable samples also showed specific differences. *Lactobacillus panis* was consistently more abundant in stable samples, whereas *Actinomyces naeslundii* was consistently more abundant in progressing samples (Fig. 1a). Also, we assessed α-diversity in the globality of samples under the three different conditions. Shannon diversity, which accounts for both abundance and evenness of the species present, was significantly higher in the fluctuating samples, followed by progressing samples and stable samples with the lower overall α-diversity (Fig. 1b). Similar results were obtained for other α-diversity indexes such as richness and Fisher α-diversity (Additional File 3, Fig. S3a). Shannon diversity values were consistently higher in the progressing and fluctuating sites than in stable sites for the study’s whole period (Fig. 1c and Additional File 3, Fig. S3b). Remarkably, fluctuating samples were the ones showing higher α-diversity, statistically significantly higher than the other two groups, and at months 8, 10, and 12 (Fig. 1c). After treatment, all conditions showed a stabilization in α-diversity (Fig. 1c).

Shannon diversity’s rate of change was higher during disease progression for most of the sampled points, while the rate of change of Shannon diversity was consistently lower in the stable sites (Fig. 1d). Thus, the periodontal treatment seems to stabilize the rate of diversity change in the progressing sites and lower it in the other two conditions (Fig. 1d).

Three different β-diversity metrics—the Jaccard index (for membership), Bray–Curtis (B.C.) dissimilarity (for abundance), and weighted-Unifrac (for phylogenetic relatedness) were used to assess bacterial communities differences. β-diversity multivariate tests yielded significant results for the progressing and fluctuating sites when compared to the stable samples, whereas progressing and fluctuating site samples were not significantly different (Additional File 4, Fig. S4).

**Fig. 2:**
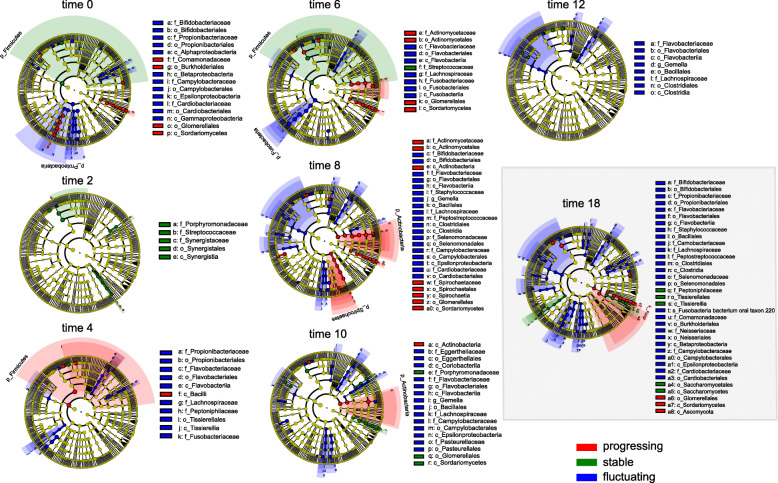
Temporal LEfSe analysis. Cladograms indicate the polygenetic distribution of taxonomic lineages within different samples at different time points as determined by linear discriminant analysis (LDA) effect size (LEfSe). Cladograms show biomarkers at different levels of taxonomic classification from the kingdom (inner circles) through phylum, class, order, family, and genus to species (outermost circles). Only taxons with LDA scores higher than two are shown. The cladogram in the gray rectangle represents the samples after periodontal treatment

Taxa associated with one of the analyzed conditions at different times were identified using the linear discriminant analysis effect size (LEfSe) [20]. Taxa with effect size (LDA) scores higher than 2 could be considered biomarkers of the different conditions. The most specific associated taxa samples were the fluctuating samples with Fusobacteria and Flavobacteria frequently associated with them (Fig. 2). Stable samples showed Firmicutes and, most specifically, the family Streptococcaceae as the most abundant phylogenetic unit, while in the case of progressing sites after the sixth-month Actinobacteria (family Actinomycetaceae) was the most abundant phylogenetic unit (Fig. 2). Surprisingly, in the samples of month 18, the biomarkers of the different communities seemed to diverge instead of converging due to periodontal treatment (Fig. 2).

### Temporal network analysis and network cartography

**Fig. 3:**
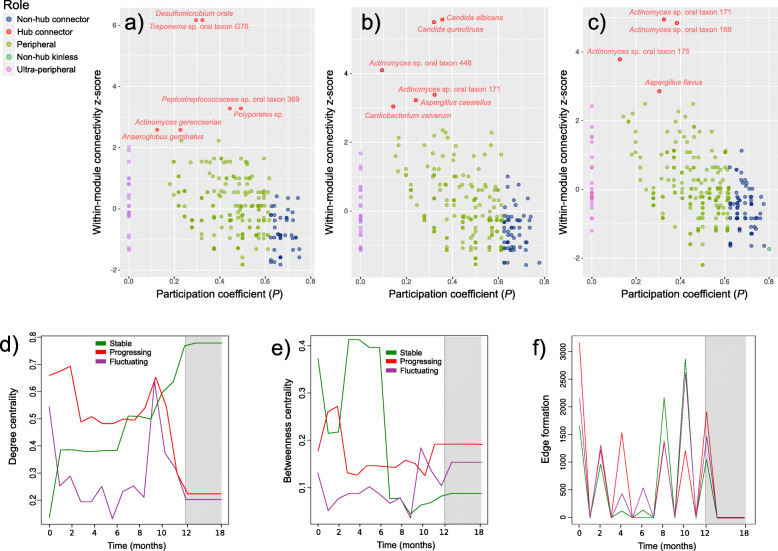
Network cartography and profiles of network centralities. **a–c** Species are assigned roles in the network. The metrics to assess the roles in the sub-community include within-module degree, which measures how well a particular species is connected to others in the same sub-community (module), and among-module connectivity, which measures how a species is linked to other modules in the network. Species are classified as hub connectors, hub kinless, non-hub connector, non-hub kinless, peripheral, provincial, and ultra-peripheral [23]. **d** Temporal evolution of degree centrality in the co-occurrence networks of the different groups. **e** Temporal evolution of betweenness centrality in the co-occurrence networks of the different groups. **f** Temporal evolution of the formation of edges in the networks of the different groups. The grey zones in **d–f** represent the period after treatment

Microbial abundances are not independent, and traditional statistical metrics (e.g., correlation) for detecting OTU-OTU relationships can lead to spurious results. Moreover, microbial sequencing-based studies typically measure hundreds of OTUs on only tens to hundreds of samples; thus, inference of OTU-OTU association networks is severely underpowered. We used SPIEC-EASI to reconstruct ecological networks. This statistical method addresses both of these issues [21]. We first performed temporal network analysis and generated dynamic networks as described in the vignette of the tsna R package [22]. We then assigned roles to the different species in the ecological network, as described by Guimerà and Amaral [23]. These authors demonstrated that nodes could be classified into universal roles according to their pattern of intra- and inter-module connections. They are thus yielding a “cartographic representation” of complex networks [23]. Within-module degree *z* measures how “well-connected” a particular node (bacterial species) is to other nodes in the same module. High values of *z* indicate high within-module connectivity and vice versa. The participation coefficient (*P*) defines how the node is positioned in its module and with respect to other modules. The participation coefficient is close to 1 if its links are uniformly distributed among all the modules and 0 if all its links are within its module [23]. Based on *z* and *P*, nodes in a network can be classified as hubs if *z* ≥ 2.5 and non-hubs if *z* < 2.5. In all three conditions, most nodes were classified as peripheral or ultra-peripheral, that is non-hub nodes with most links within their modules (0.05 < *P* ≤ 0.62) [23] (Fig. 3a–c). In stable sample networks, *Actinomyces gerencseriae*, *Anaeroglobus geminatus*, *Desulfomicrobium orale*, Peptostreptococcaceae bacterium oral taxon 369, Polyporales sp., *Treponema* sp. oral taxon G76, acted as hub connector; that is, hubs with many links to most of the other modules (0.30 < *P* ≤ 0.75). In the progressing sites, *Actinomyces* sp. oral taxon 171, *Actinomyces* sp. oral taxon 448, *Aspergillus caesiellus*, *Candida albicans*, *Candida quercitrusa*, and *Cardiobacterium valvarum* were hub connectors (Fig. 3a, b, Additional File 5, Table S1). Finally, in fluctuating sites, *Actinomyces* sp. oral taxon 169, *Actinomyces* sp. oral taxon 171, *Actinomyces* sp. oral taxon 175, and *Aspergillus flavus* acted as hub connectors (Fig. 3c, Additional File 5, Table S1).

We then performed a temporal analysis of two network centralities: degree and betweenness centrality. The degree of a node (or a species) refers to the number of links to other interacting partners in the network, while the betweenness of taxa is a measure of taxa’s control in the network. Stable samples showed an increase in degree centrality throughout the study (Fig. 3d) while progressing sites showed a high degree of centrality consistently until month 10 when there was a steep decrease. Interestingly, fluctuating sites showed low-degree centrality compared to the other two conditions until the tenth month when, as in the progressing sites, a steep increase occurred (Fig. 3d). High betweenness centrality implies that a corresponding node has more influence in the network and vice versa. In betweenness centrality, progressing and fluctuating sites showed a similar pattern to the one observed for degree centrality, low values in fluctuating sites, and higher in progressing sites (Fig. 3e). Stable samples showed, in general, sharper oscillations, with a high peak between 2 and 6 months (Fig. 3e). In all three cases, there was a significant increase in-betweenness after treatment, special pronounce in the case of the progressing sites (Fig. 3e). Finally, we measured the number of edges formed at different times in the different temporal networks. Progressing sites showed the most distinct profiles with a high peak at month 4 and a lower number of edges formed at month 8 (Fig. 3f). At months 4 and 6, the stable networks showed a significantly lower formation of nodes than progressing and fluctuating sites (Fig. 3f).

### Temporal community dynamics and dominance structure vary with clinical progression

**Fig. 4:**
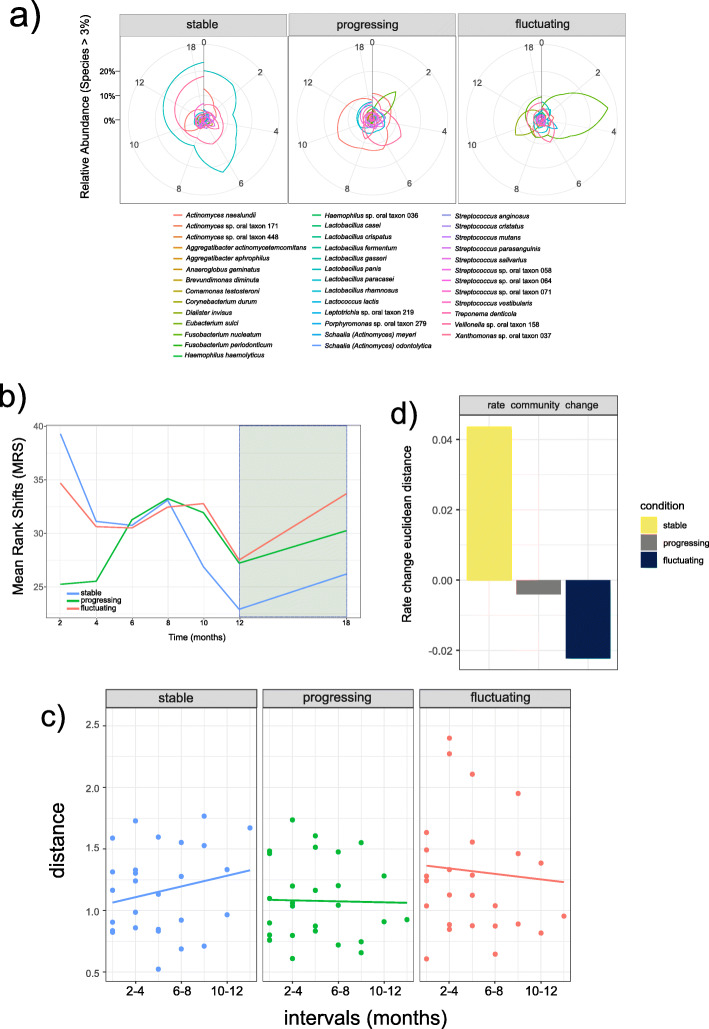
Temporal diversity indices. **a** Rank clocks highlighting the reordering in the relative abundance of dominant species in stable, progressing, and fluctuating sites. We selected the species with more than 10% abundance at least one-time point (see Fig. 1c). Vertical black bars show the starting “12 o’clock” position on the rank clock. **b** Evolution of mean rank shifts (MRS) representing the relative changes in species rank abundances. **c** Rate of community change calculated as differences in species composition between samples at increasing time intervals. Differences in species composition are characterized by Euclidean distances, calculated on pairwise communities across the entire time series. If the distance between samples does not change as time-lags increase, then the community is stable. If sample distance increases over time, the community is unstable and undergoing directional change. If sample distance decreases over time, then the community is unstable and undergoing convergence27. **d** Bar-plot of the values of the slopes of the rate community change

To better understand the dynamics in microbial species composition within and across sample types, we first visualize the degree to which the most abundant species reorder over time and the effect of periodontal treatment on this rearrangement of species. There were substantial shifts in species dominance in all communities over the sampling period. Rank clocks highlight that there has been high reordering in the relative abundance of the dominant species in the fluctuating and progressing microbiomes, but to a less extent in the stable communities (Fig. 4a).

The temporal measure of species reordering measured as Mean Rank Shifts (MRS) showed large oscillations during the study period. MRS progression describes relative changes in species rank abundances and indicates the degree of species reordering between two time points. Calculating mean rank shifts highlights that communities’ stability diverged from the beginning of the study, with changes an increase of MRS at the beginning of the study in progressing sites and a decrease during the same period (2 to 6 months) in stable and fluctuating sites (Fig. 4b). At month 8, there was a peak in all the groups, after which all of them showed a steep decrease, especially pronounced in the stable samples (Fig. 4b). Interestingly, periodontal treatment resulted in an MRS increase in all groups (Fig. 4b).

We also assessed the rate and pattern of variability within a community, which indicates whether species reordering over time results in a directional change; that is, sample distance increases over time. Differences in species composition were characterized by Euclidean distances, calculated on pairwise communities across the entire time series. The regression line’s slope indicates the rate and direction of compositional change in the community [24]. Communities converge if sample distances decrease over time, whereas if sample distance increases over time, the communities are undergoing directional change [24]. The stable samples showed a more significant directional change through time than the progressing and fluctuating samples (Fig. 4c). The progressing and fluctuating communities were unstable with a negative slope and undergoing convergence, with the fluctuating samples showing a more negative value of the slope (Fig. 4c, d).

### Defined sub-communities followed marked temporal fluctuations

Next, we considered the degree to which different species clustered in their abundance profiles during the study. Using an infinite Gaussian process mixture model [25], we found that in the case of the stable samples, there were 12 clusters of behavior, while in the progressing and fluctuating groups, there were 11 and 13, respectively (Additional File 6, Fig. S5 and Additional File 7, Table S2). One cluster had the most species in all three conditions, more than 200 (cluster 2). More importantly, these clusters shared a large proportion of species. One hundred eighty-three species were common to all three clusters 2 (Additional File 6, Fig. S5d).

Furthermore, although the composition of these clusters was similar, their behavior was very different. For example, cluster 2 from fluctuating samples and the other two groups followed opposite trajectories at the beginning of the study, but after month 8, while stable samples showed a decrease in the abundance of cluster 2, progressing sites maintained a high proportion of those bacteria (Additional File 6, Fig. S5e). On the other hand, cluster 2 of the fluctuating samples followed a completely different profile. While in high proportion up to month 6, it decreased in proportion at the end of the study (Additional File 6, Fig. S5e).

**Fig. 5:**
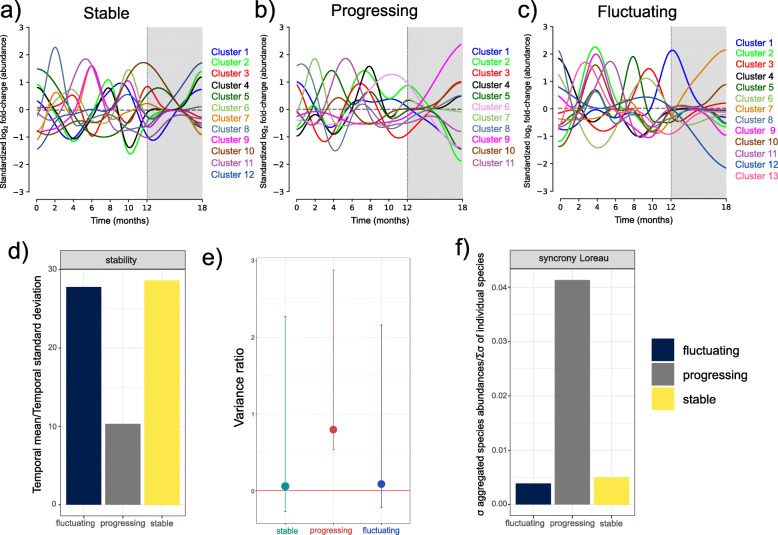
Stability of the oral microbiome communities in the three different conditions. **a–c** Dirichlet process Gaussian process mixture model (DPGP) clusters of microbial species. Clusters are standardized to log2 fold-change of abundance. Colors and cluster numbers are arbitrary. The actual composition of the different clusters is presented in Additional File 8, Table S3. The grey zone represents the period after treatment. **d** Community stability aggregates species abundances within replicate and period and uses these values to calculate community stability as the temporal mean divided by the temporal standard deviation [26]. **e** Variance ratio calculated as the ratio of the community’s variance as a whole relative to the sum of the individual population variances. Bars represent the 95% confidence intervals after 1000 bootstraps. **f** Loreau and de Mazancourt synchrony metric comparing the variance of aggregated species abundances with the summed variances of individual species [27]

One unexpected result was obtained when we plotted all clusters on the same graph, and temporal fluctuations of the different clusters were observed, in what looks like a succession of different communities, with peaks and valleys of abundances (Fig. 5a–c). What is more, in some cases, two or more clusters shared temporal peaks but behaved differently at other times. Thus, the periodontal treatment seemed to work by selecting specific clusters while reducing others in abundance (Fig. 5a–c). In particular, cluster 2 was relatively less abundant in progressing and fluctuating samples but no stable samples (Fig. 5a–c).

### Progressing samples are characterized by high synchrony, where different species respond similarly through time

One key question in the relationship between species diversity and stability is how the community’s components affect the whole community’s aggregate properties’ stability. Unstable species populations may still maintain stable communities, which is a time series, is reflected by a pattern in which species negatively covary whereas total community stability remained relatively stable. The previous section showed that different communities followed a succession in time, with some clusters following similar or opposite profiles (Fig. 5a–c).

To assess community stability, we used Tilman’s method to aggregate species abundances within replicate and time and utilize these values to calculate community stability as the temporal mean divided by the temporal standard deviation [26]. As expected, the stable communities reported higher stability values, as defined by Tilman [26], than progressing communities (Fig. 5d). Surprisingly, fluctuating sites were only slightly less stable than the stable communities (Fig. 5d).

The variance ratio (V.R.) was one of the first metrics to characterize species covariance patterns [28]. It was used in an early synthesis paper of species covariance in long time series [29]. The metric compares the community’s variance as a whole relative to the sum of the individual population variances. If species vary independently, then the variance ratio will be close to 1. A VR < 1 indicates predominately negative species covariance, whereas a V.R. > 1 indicates that species generally positively covary. Our results show that in the species of the stable samples, covary predominantly negatively (V.R. < 1), whereas in the progressing and fluctuating samples, they do positively (V.R.> 1) (Fig. 4e). A significant criticism of the variance ratio is that it is sensitive to species richness, which is of particular concern when the metric is used to compare communities with different species richness levels. Other alternative metrics that quantify species asynchrony have been developed in part to respond to this issue. We measured synchrony using the Loreau and Mazancourt metric that compares the variance of aggregated species abundances with the summed variances of individual species [27]. This metric ranges from 0 (perfect asynchrony between species) to 1 (perfect synchrony). Stable and fluctuating samples presented a lower synchrony level in the communities than the progressing (Fig. 5f).

### Progressing and fluctuating sites follow a neutral model of phylogenetic recruitment of new species

**Fig. 6:**
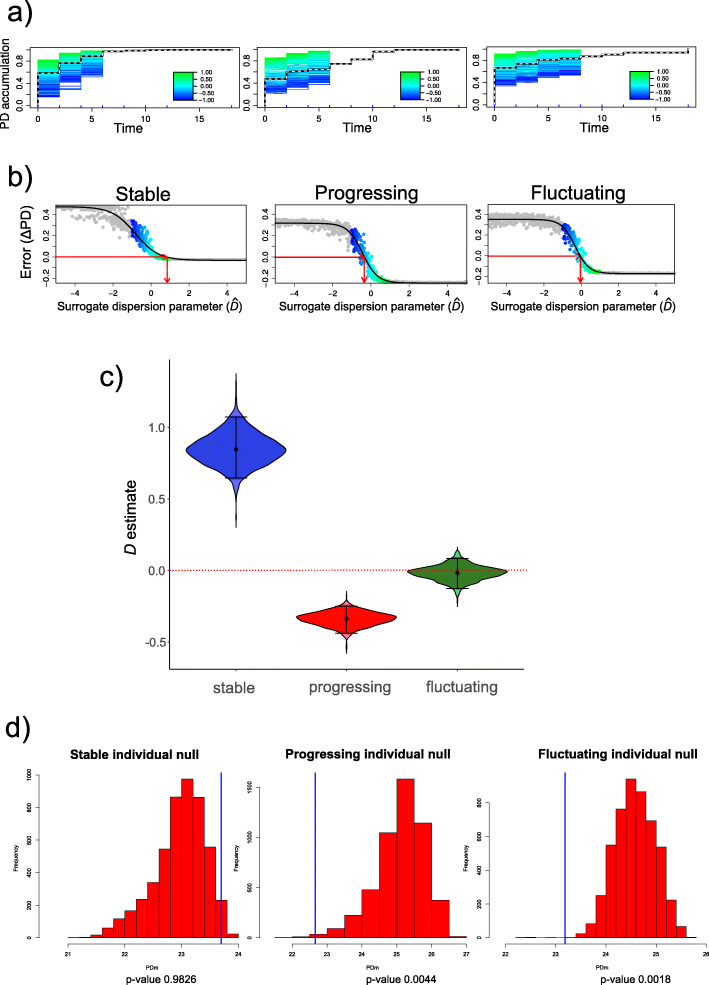
Phylodiversity accumulation and model fitting in the three datasets. **a** Empirical and surrogate phylodiversity accumulation curves. Surrogate curves are colored according to the *D* value. New species with a previously detected close relative contribute little phylodiversity and cause slow phylodiversity accumulation (blue). New species that do not have a close relative contribute more phylodiversity and cause faster accumulation (green). **b** Empirical and surrogate data are compared to generate an estimate for *D*. The red arrows show the process of error minimization, yielding a *D* estimate. **c** Dispersion parameter (*D*) estimates. The dot in the center of a violin is the mean, and bars represent 95% confidence intervals for the *D* estimate. **d** Testing against alternative “individual null.” The “individual null” is an alternative null model that accounts for the relative abundance of OTUs in the data subset

Knowing when and why new species are recruited into microbial communities has significant implications in understanding the dynamics of health and progression and implications in devising strategies to managing the microbiome to restore a healthy status. We used a mathematical model developed by Darcy et al. that describes the order in which new species are detected in microbial communities over time within a phylogenetic framework [30]. The model estimates the degree to which the recruitment of new species is more or less likely when a close relative has been previously recruited. The model estimates an empirical dispersion parameter *D*, which quantifies the degree to which first-time species detections are phylogenetically related. If *D* ≠ 0, then species are preferentially added if they have relatively low (*D* < 0) or relatively high (*D* > 0) phylogenetic distance to the resident community, yielding accumulations of total phylodiversity that are relatively slow (*D* < 0) or relatively fast (*D* > 0) compared with the neutral model (*D* = 0) [30]. Figure 6a shows the phylogenetic accumulation of the three datasets. New species with a previously detected close relative contribute little phylodiversity and cause slow phylodiversity accumulation (blue). New species that do not have a close relative contribute more phylodiversity and cause faster accumulation (green). Stable samples showed a relatively faster accumulation of phylogenetic diversity than the neutral model (green fraction is reduced with time). In contrast, the progressing and fluctuating samples followed the neutral model of phylogenetic accumulation (green and blue fractions do not change with time) (Fig. 6a). Figure 6b shows the estimates for *D*’s empirical value, which is the value at which ΔPD = 0. Figure 6c shows the distribution of *D* estimates where dots within violins are means. While stable samples show mean estimates of *D* much higher than 0, new species are more likely to be recruited if they are phylogenetically distant (overdispersed) from previously recruited species, progressing and fluctuating sites followed a neutral model, *D ≤* 0.

The “individual null” test results confirm the previous conclusions; progressing and fluctuating sites followed a neutral model of phylogenetic recruitment of new species (Fig. 6d). This “individual null” accounts for differences in total species relative abundance across a time series, while the simulation above only considers the presence-absence of species.

## Discussion

The objective of the present study was to begin to understand changes in the oral microbiome’s community structure by examining its long-term dynamics over time, something that has never been done, particularly in the breadth and depth as presented here. Here we performed a time-series analysis of the oral microbiome in people with periodontal disease, focusing on the ecological elements that could explain the transition from health to disease rather than changes in species composition. Our findings highlight the importance of time-series data to facilitate ecological explanations of how microbiome stability comes about over time. For 1 year, we followed a defined cohort and sampled the same sites from the same patients. From those patients, we sampled the following: sites that showed periodontitis progression, sites that did not change clinically during our study, and sites that fluctuated, exhibiting a cyclic deepening followed by spontaneous recovery to their original depth [12, 13]. Because we followed the same teeth from the same patients and the three kinds of samples had the same clinical features at the baseline of the study, this cohort provided an unparalleled opportunity to identify what changes in the community could be associated with the progression of the disease.

As expected, the microbial composition of the different groups varied largely throughout the study. Although cross-sectional studies have found an association of individual organisms with the disease, the oral microbiome’s relative composition varies from study to study [31–33]. Our results show that the microbial communities in our samples followed oscillating patterns significantly different in the three clinical groups studied. Oral sites from patients with periodontitis with no clinical signs of disease are already impacted and are already different from what is considered health [34]. Interestingly, after treatment, although the clinical status of the three groups improved and α-diversity consistently stabilized or slightly decreased, the different microbiomes did not converge into a “core” healthy microbiome shared by all types of samples. However, they differ from each other, and they were more similar to the last samples before treatment, pointing to the interpersonal variability [35]. Periodontal treatment leads to stabilizing or decreasing the rate of change of α-diversity and narrowed the frequencies of the different clusters around the mean but had no effect in selecting a “healthy” core microbiome.

Measures of diversity showed a consistent higher α-diversity in fluctuating sites, and as reported in other studies, Shannon diversity was significantly higher in progressing samples [36, 37] than in stable sites. Thus, although it has been proposed that higher diversity may lead to higher stability of the microbial community [38], the relationships between diversity and stability are much more nuanced, and high diversity is not always a sign of health [39].

We further hypothesized that the community’s emergent properties, such as stability and robustness, could be a complement to defining dysbiosis in the oral cavity during disease progression. Dysbiosis has also been interpreted as a breach of the microbiome’s robustness and a transition to a new, more unstable state [40]. However, determining which factors make specific microbiota robust is a difficult task. We observed that the communities in the three groups had a different network topology. Temporal network analysis of these communities showed species that acted as hub connectors, hubs with many links to most of the other modules, of the networks in all three conditions. Although in stable samples, these hub connectors were represented by a variety of species in the progressing and fluctuating sites, fungi and species of the genus *Actinomyces* represented the vast majority of these hubs. In the skin and lung microbiome of humans, it has been suggested that fungi play a stabilizing role in the organization of the ecological network [41]. Whether that is the role in the progressing and fluctuating sites is not known yet.

Microbial hubs are strongly interconnected and have a severe effect on communities. These nodes are essential for maintaining network structure and potentially important species for community structure [42, 43]. Although correlation does not imply causation, the removal of network hubs can cause a drastic shift in a microbiome’s composition and functioning [44]. The higher the centrality values, the more associations between community members are established and, consequently, the more (functional) redundancy exists [45]. Lower average degree centrality has been observed when associations between community members are not as tight [45]. Despite the wealth of theoretical and fieldwork, there is still no complete agreement on the complexity–stability relationship in ecosystems. Stable networks were characterized by increased degree centrality and a high betweenness centrality during the first half of the study, implying an increase in complexity and functional redundancy in the stable communities. Progressing samples showed consistently higher centrality (degree and betweenness) than fluctuating samples until the tenth month. Edge formation profiles indicated that the period between 2 and 6 months is essential in restructuring the different networks.

Many community structure measures, such as diversity indices, do not capture ecological systems’ temporal dynamics. Reordering describes relative changes in species rank abundances in time. We found a high reordering in the relative abundance of the dominant species in the fluctuating and progressing, and lower in the stable communities. More importantly, we identified a series of bacterial clusters that followed similar relative abundance patterns along the period of study. The microbiome composition of these clusters was specific to the sampling group. However, we identified a large “core” cluster shared by the three groups, albeit it followed different dynamics depending on the sample type.

Interestingly, these clusters did not maintain a steady kinetic but showed a high degree of compositional fluctuations, with temporal peaks and valleys represented by different microbial clusters. These kinds of temporal fluctuations in community members have been previously described in various human microbiome sites [46, 47]. These fluctuations play a significant part in species’ coexistence in other biological systems and the stability and implications for disease states [48]. Not surprisingly, measures of stability were higher in the stable samples but also in fluctuating sites. More interesting were the results we observed when we analyze the synchrony of the observed fluctuations. Ecological synchrony refers to the level of populations that oscillate up and down together or precisely 180° out of phase. Stable samples showed a higher degree of asynchrony. Although it is a very different system, in plant ecology, synchrony of species increases local communities’ stability, and asynchrony among local communities enhanced metacommunity stability by a wide range of magnitudes [14]. Our results indicate that in the oral microbiome, the microbial populations’ temporal synchrony also controls the community’s level of stability, with higher synchrony linked to unstable communities.

Finally, we studied whether these fluctuations followed different taxa recruitment patterns, e.g., the order in which new species are detected in microbial communities over time within a phylogenetic framework [30]. The type of phylogenetic recruitment of microbial communities is critical if we want to better exploit disturbance as a tool for managing microbial systems related to human health and disease. The human microbiome generally follows the under-dispersion hypothesis. In other words, new species are more likely to be recruited if a close relative has been recruited previously (nepotism). However, the exceptions are oral communities [30]. In our study, the stable samples followed the pattern observed by Darcy et al. for oral communities; new species were more likely to be recruited if a close relative was not recruited previously. These could be explained by competition between closely related species. However, in the progressing and fluctuating sites, the recruitment of new species followed a neutral model where recruitment likelihood is not related to phylogenetic relationships among species, potentially indicating cooperating networks, which could be efficient but are often unstable [49].

## Conclusions

In conclusion, the oral microbiome’s dysbiotic process leading to the outcome of a site in patients with periodontitis seems to be determined by the community’s emergent properties, such as the level of asynchrony and the type of phylogenetic recruitment the community exhibits rather than by changes in composition. We recognize that this is a small sample size for any specific predictions to be made and that additional, more extensive studies need to be performed. Moreover, we still lack a functional/mechanistic explanation of microbiome composition that confers stable, or even transferable, metabolic phenotypes when subjected to particular diseases or environmental factors. Moving forward, more time-series studies should be performed to confirm our results, and functional studies should be incorporated to complement metagenomic analysis.

## Methods

### Experimental model and subject details

#### Human cohort

The present study subjects were recruited as part of a multi-center clinical trial to determine biomarkers of periodontal disease progression (Biomarkers of Periodontal Disease Progression Clinical Trials.gov ID NCT01489839). Subjects were monitored clinically for up to 1 year every 2 months to detect periodontal sites and subjects with periodontal disease progression. A decision was made to collect samples every 2 months to obtain a significant number of samples for time-series analysis and, at the same time, minimize the effect that the sampling process could have on the results. Pivotal studies on the topic of periodontitis progression employed a similar design [12, 50–53]. Subgingival microbial samples were collected from up to 32 sites per subject per visit. These 32 sites represent the interproximal sites of all existing posterior teeth (tooth sites 1 and 3 circled and red dots in the figure). The rationale for sampling these sites was that disease progression occurs most frequently at proximal sites on posterior teeth. Only posterior teeth of periodontitis patients were sampled. Progressing sites were selected based on the existence of microbial samples (sites 1 and 3 of posterior teeth, i.e., mesial and distal sites of pre-molars and molars). The study was described thoroughly to all subjects before obtaining informed consent. Inclusion criteria were as follows: age > 24 years; ≥ 20 natural teeth (excluding third molars); at least four teeth with at least one site of pocket depth (P.D.) of 5 mm or more and concomitant clinical attachment loss (CAL) greater than or equal to 2 mm; radiographic evidence of mesial or distal alveolar bone loss around at least two of the affected teeth; and in good general health. Exclusion criteria were as follows: current cigarette smokers; pregnant or nursing; received antibiotic or periodontal therapy in the previous 6 months; any systemic condition potentially affecting the course of periodontal disease (for example, diabetes or AIDS); chronic use of non-steroidal anti-inflammatory drugs; or any condition requiring antibiotic coverage for dental procedures. In total, a subset of 15 patients was selected for this study. Disease progression was defined based on the evolution of clinical attachment loss (CAL) as described in Teles et al. [13]. Linear mixed models (LMM) were fitted to longitudinal CAL measurements for each tooth site, and the predicted CAL levels were used to categorize sites regarding progression or regression. The threshold for progression was established based on the model estimated error in predictions. Three groups of sites studied were defined based on the LMM results [15]. The three groups studied were as follows: sites that showed progression of the disease, sites that remained stable during the study, and sites that fluctuated, exhibiting a cyclic deepening followed by spontaneous recovery. Clinical parameters defining the subjects are presented in Table S4. Participants received periodontal therapy (scaling and root planning, SRP) at the end of the study and were monitored at 3 and 6 months. Sampling was only performed at month 6 after treatment to assure the oral microbiome was not influenced by periodontal treatment. Power analysis was performed using the R package “micropower” [16].

#### Subgingival plaque samples

Two plaque samples were taken from each posterior tooth’s mesial and distal aspects (excluding third molars) for up to 64 samples. After removing supragingival plaque, subgingival plaque samples were individually collected from each site with a single stroke using a sterile Gracey mini-curette. After collecting one plaque sample, the sterile end of another curette collected the following sample. Each sample was placed in a separate microcentrifuge tube.

#### Ethics statement

Subject recruitment and study procedures were approved by and carried out in accordance with the Institutional Review Board at The Forsyth Institute.

### Nucleic acid extraction and 16S rRNA and ITS sequencing

Total genomic DNA was extracted as described elsewhere [54]. PicoGreen was used for DNA quantification, and a 16 s rRNA metagenomics library was performed using Swift Amplicon 16 s + ITS Panel kit. This kit provides a single primer pool covering all the variable regions of the 16S rRNA gene (V1–V9) and fungal ITS1 and ITS2 genes. In total, 50 pg of gDNA was used for multiplex PCR in a 20 μl reaction mix. The PCR condition consisted of 30-s incubation at 98 °C followed by first five cycles of 98 °C, 10 s; 63 °C, 5 min; and 65 °C, 1 min; then 26 cycles of 98 °C, 10 s; 64 °C, 1 min; and 65 °C, 1 min. The multiplex PCR product was purified with AMPure® XP Beads. After two, around 80% ethanol washes, eluted with 5 μl of i5 index, 10 μl of i5 index, and 35 μl of Indexing Reaction Mix. The indexing PCR was performed by incubating at 37 °C for 20 min. The indexing PCR was cleaned with an adding ratio of 0.85 PEG NaCl into Indexing PCR. The individual library was quantified using the KAPA library quantification kit (Kapa Biosystems, catalog number: KK4824) and monitoring on the BioRad CFX 96 real-time PCR system. Barcoded samples were pooled equimolarly for sequencing one MiSeq 2 × 250 cycle run. The library preparation was performed at the Gene Expression & Genotyping of the Interdisciplinary Center for Biotechnology Research (University of Florida). The MiSeq run was performed at NextGen of the Interdisciplinary Center for Biotechnology Research (University of Florida).

### Taxonomic profiling

When possible, in all bioinformatics analyses, GNU parallel was used [55]. Sequences were filtered for quality using Trimmomatic [56]. Once filtered, sequences were merged using bbmerge [57]. Next, chimeras were removed using USEARCH [58] against the SILVA database [59]. Phylogenetic assignment and relative quantification were performed using Kraken2 [18] and Bracken [19] against a custom 16S rRNA dabatase for the oral microbiome extracted from the HOMD database [60] and the UNITE database [61] for fungal ITS sequences. We generated a custom database of the ITS sequences with selected fungi species that had been previously identified in oral samples and the ITS sequences of *Entamoeba gingivalis*. Taxonomic representation of statistically and biologically consistent differences between the different groups was performed using the linear discriminant analysis effect size (LEfSe) method [20].

### Diversity measurements

#### α-Diversity

Total α-diversity and ANOVA Shannon of the time series were measured using the package “microbiome” [62] and “microbiomeSeq” [63]. To examine how Shannon diversity and its rate of change vary across time, we performed volatility analysis using QIIME2 [64, 65].

#### Ordination and β-dispersion

Multidimensional clustering was performed using PCoA with three different dissimilarity distances: Bray-Curtis, Jaccard, and weighted-Unifracusing. Ordination analysis was performed using the R package “microbiome” [62]. Counts were normalized using as compositional. β-dispersion was calculated by computing the average distance of individual groups to the group centroid. Finally, permutation analysis of variance (PERMANOVA) was calculated on the β-dispersion between all possible pairwise combinations of the grouping variable levels.

### Time-series analysis

#### Forecasting analysis of clinical parameters

Dickey-Fuller test of stationary was performed using the adf.test function of the “tseries” R package [66]. Time series were analyzed using the “aTSA” R package [67]. We applied the ARIMA model for stationary series directly. We transformed the series into stationary on the values for non-stationary series by subtracting *CAL*_*t-1*_ from *CAL*_*t*_ for all values *t*. This technique is called differencing and can be done with the “diff” function of the aTSA package.

#### Bar-plot species

Species composition of the different groups and time points were represented as bar-plots using the package “phyloseq” [68].

#### Inference and cartography of ecological networks

To reconstruct ecological networks from data abundance, we used SPIEC-EASI [21], a statistical method for the inference of microbial ecological networks from amplicon sequencing datasets. We then performed a cartographic representation of the networks based on the connectivity of the nodes as described by Guimera and Nunes Amaral [23]. The cartographic representation was obtained using the function “netcarto” from the R package “rnetcarto” [69].

#### Temporal network analysis

To visualize temporal changes in the structure of ecological networks, we performed temporal network analysis with the R package “tsna” [22]. First, we generated a network as a static edge list with its associated vertex attributes. We then import the temporal data associated with the dynamic edges and dynamic nodes. We then added the temporal data to the static network we created above to form a dynamic network, using the networkDynamic() function. Finally, we measured edge formation over time on this dynamic network and calculated the network’s rolling betweenness and degree centralization using the function tSnaStats().

#### Dynamics of species over time

To visualize the degree to which species reorder over time, we used rank clocks, calculate the relative changes in species rank abundances or mean rank shifts (MRS), and assess the rate and pattern of variability within a community, which indicates whether species reordering over time is resulting in a directional change we used the R package “codyn” [70].

We clustered the different species’ trajectories using the Dirichlet process Gaussian process mixture model (DPGP) software [25]. A Dirichlet process determines the number of clusters in a non-parametric manner, while a Gaussian process models the trajectory and time dependency of the specific species in a non-parametric manner.

Measurements of stability, variance ratio, and synchrony were performed using the R package “codyn” [70]. Finally, we used the phylogenetic model for the recruitment of species described in Darcy et al. [30] to test whether communities followed a neutral recruitment model of new species. The model indicates whether new species are more likely to be recruited if a close relative has been recruited previously (nepotism) or whether new species were more likely to be recruited if a close relative was not recruited previously. The statistical model describes the probabilities of detecting new species over time. First, the model is used with empirical data via simulations, where empirically detected species are resampled using the model with known parameter values to produce surrogate datasets. To this goal, the model’s dispersion parameter (*D*) is fixed and recorded, determining the extent to which species with a close relative are preferentially added to the surrogate community (or, conversely, if species without a close relative are preferred). Next, the parameter estimation compares the empirical pattern of species detection to the surrogate datasets (which have known *D* values) to determine which *D* value best describes the empirical data. Finally, the hypothesis testing compares empirical data to repeated simulations under the neutral model, which is *D* = 0, and all species have the same probability of being detected for the first time.

## Supplementary Information

**Additional File 1: Figure S1.** Experimental design. a) 15 participants were selected from a total cohort of 415 participants. These patients had all three conditions we wanted to study in their mouths. Thus genetic background should have a minimal effect on the outcome of individual sites. At baseline, all teeth used were clinically identical. Samples of subgingival plaque were taken every two months for one year, after which all patients underwent scaling and root planing as treatment. After three months for a visual check-up and again after six months when they were monitored, all of them came back to the clinic, and samples were also taken. b) Desired trajectories of sites sampled. Stable sites: clinical attachment loss (CAL) remained unchanged during the study. Progressing sites: CAL increased steadily and significantly during the study. Fluctuating sites: exhibited a cyclic deepening followed by spontaneous recovery, with no defined outcome. c) Different stages of periodontitis progression. (i) The first stage (gingivitis) occurs when calculus builds up and gums are inflamed. At his stage can reverse to health. (ii) If gingivitis is untreated dental plaque turns into hard tartar, and regular oral hygiene is not enough to treat it. Inflammation causes the gum to separate from the tooth, forming pockets. This stage is known as periodontitis, and it is moderately severe. (iii) The last stage represents an irreversible form of gum disease with severe bone loss, deep pockets, and the danger of losing the tooth.

**Additional File 2: Figure S2.** Time-series forecasting of clinical attachment loss (CAL) based on the observed results. a) Observed results of CAL in the stable samples before treatment. b) ARIMA forecast results for the stable sites. c) Observed results of CAL in progressing samples before treatment. d) ARIMA forecast results for the progressing sites. e) Observed results of CAL in fluctuating samples before treatment. f) ARIMA forecast results for the fluctuating sites. Grey zones represent 95% confidence intervals. Blue zones represent the period after periodontal treatment.

**Additional File 3: Figure S3.** Αlpha-diversity results. a) α-diversity of all samples in the three different groups measured as richness and Fisher index. b) ANOVA results for Shannon diversity comparisons. Levels of significance (p < 0.05, p < 0.01, p < 0.001) were marked by one, two and three asterisks, respectively.

**Additional File 4: Figure S4.** β-diversity of microbial communities. Multidimensional clustering was performed using PCoA with three dissimilarity distances: a) Bray-Curtis, b) Jaccard, and c) weighted-Unifrac. β-dispersion was calculated by computing the average distance of individual groups to the group centroid. Permutation analysis of variance (PERMANOVA) and corresponding r-squared and p-values are calculated on the β-dispersion between all possible pairwise combinations of the grouping variable levels.

**Additional File 5: Table S1.** Taxa’s role in the network cartography as shown in Fig. 3.

**Additional File 6: Figure S5.** Dirichlet process Gaussian process mixture model (DPGP) clusters in species abundance trajectories. a) Clusters corresponding to stable samples. b) Clusters corresponding to progressing samples. c) Clusters corresponding to fluctuating samples. d) Venn-diagram of cluster 2, the largest cluster, shows a large group of organisms shared by the three groups from the three different groups. e) Trajectory of cluster 2 in the three groups.

**Additional File 7: Table S2.** Species composition of clusters presented in Fig. 5.

**Additional File 8: Table S3.** Clinical measurements of the sites used in the study.

**Additional File 9: jupyter notebook**. The notebook contains all code and software used to process and analyze the data are available as a fully reproducible computing environment in the jupyter notebook, which is a .ipynb file that can be launched typing the command *jupyter notebook* on a terminal on the directory that contains the notebook. The software needed can be installed using pip (pip install notebook) or conda (conda install -c conda-forge notebook).

## Data Availability

The datasets generated and/or analyzed during the current study are available in the Sequence Read Archive (SRA) data repository of NCBI with submission ID SUB9017720 and BioProject ID PRJNA725874 (https://www.ncbi.nlm.nih.gov/sra/?term=PRJNA725874) [71]. In addition, all code and software used to process and analyze the data are available as a fully reproducible computing environment in the Additional File 9, jupyter notebook provide in the supplementary materials.
