# Supplementary figures and images for: Long-term dynamics of the human oral microbiome during clinical disease progression
Source: BMC Biol. 2021 Nov 6;19:240. doi: 10.1186/s12915-021-01169-z (PMC8572441; doi:10.1186/s12915-021-01169-z)

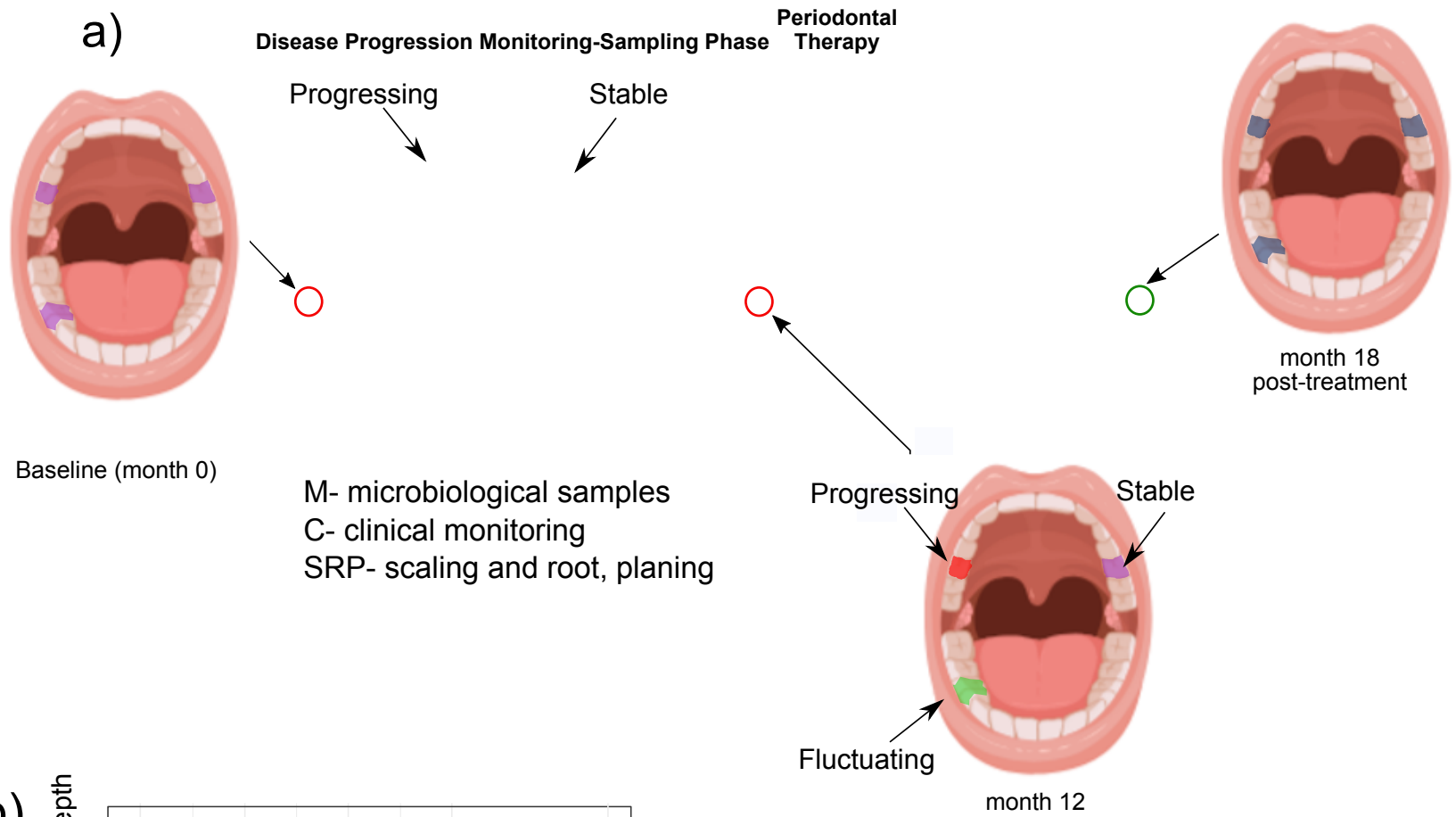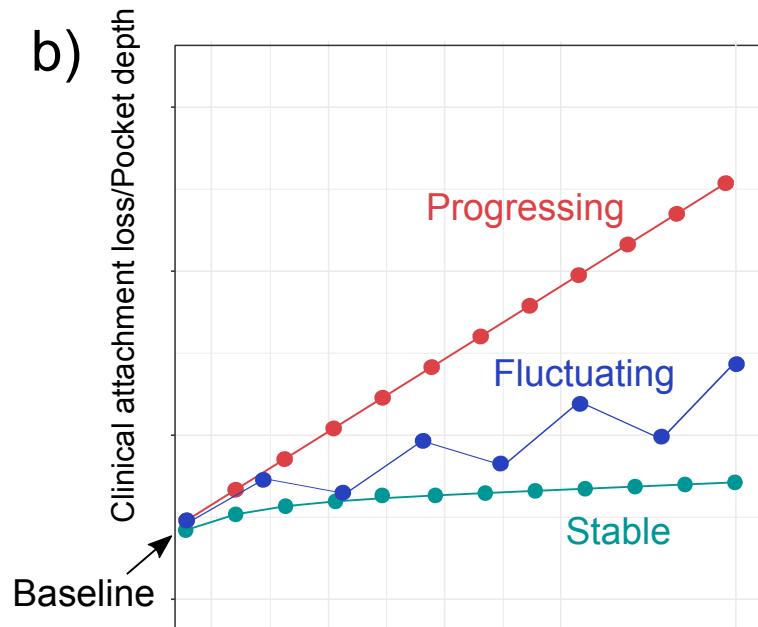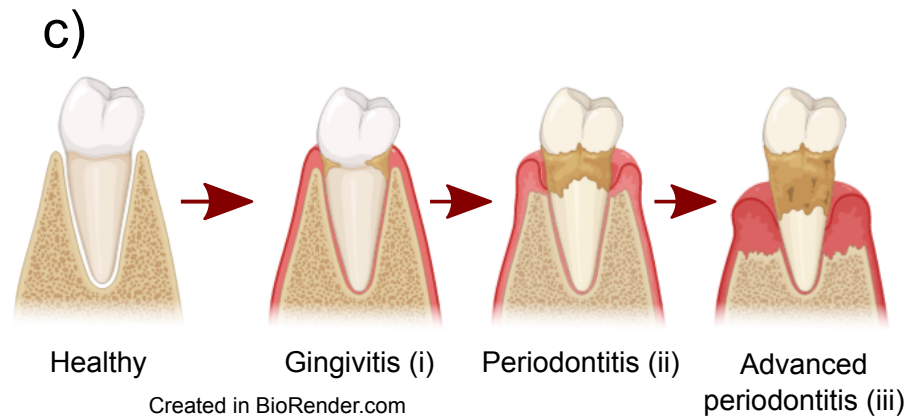

Supplement: Supplementary file 1 — Additional File 1: Figure S1. Experimental design. a) 15 participants were selected from a total cohort of 415 participants. These patients had all three conditions we wanted to study in their mouths. Thus genetic background should have a minimal effect on the outcome of individual sites. At baseline, all teeth used were clinically identical. Samples of subgingival plaque were taken every two months for one year, after which all patients underwent scaling and root planing as treatment. After three months for a visual check-up and again after six months when they were monitored, all of them came back to the clinic, and samples were also taken. b) Desired trajectories of sites sampled. Stable sites: clinical attachment loss (CAL) remained unchanged during the study. Progressing sites: CAL increased steadily and significantly during the study. Fluctuating sites: exhibited a cyclic deepening followed by spontaneous recovery, with no defined outcome. c) Different stages of periodontitis progression. (i) The first stage (gingivitis) occurs when calculus builds up and gums are inflamed. At his stage can reverse to health. (ii) If gingivitis is untreated dental plaque turns into hard tartar, and regular oral hygiene is not enough to treat it. Inflammation causes the gum to separate from the tooth, forming pockets. This stage is known as periodontitis, and it is moderately severe. (iii) The last stage represents an irreversible form of gum disease with severe bone loss, deep pockets, and the danger of losing the tooth. [file 12915_2021_1169_MOESM1_ESM.pdf]

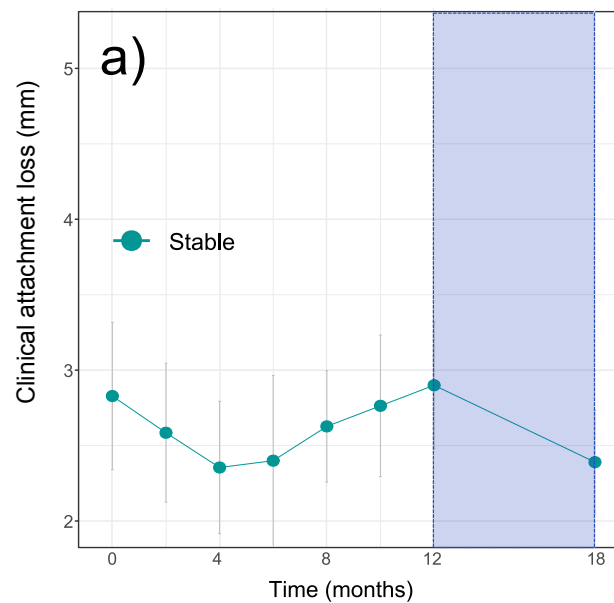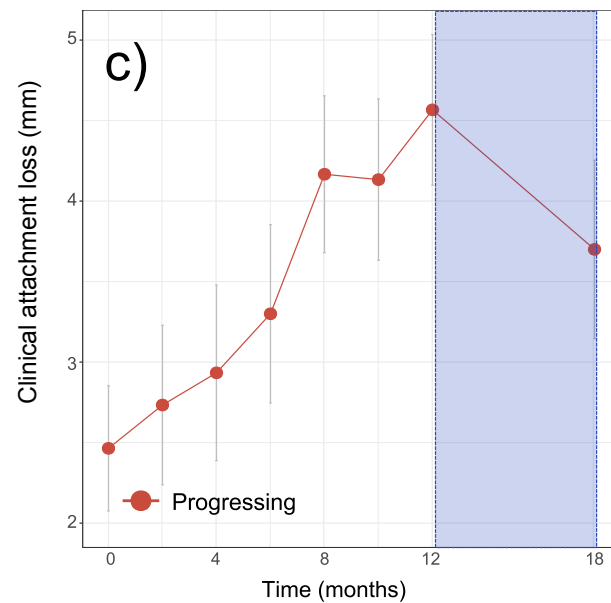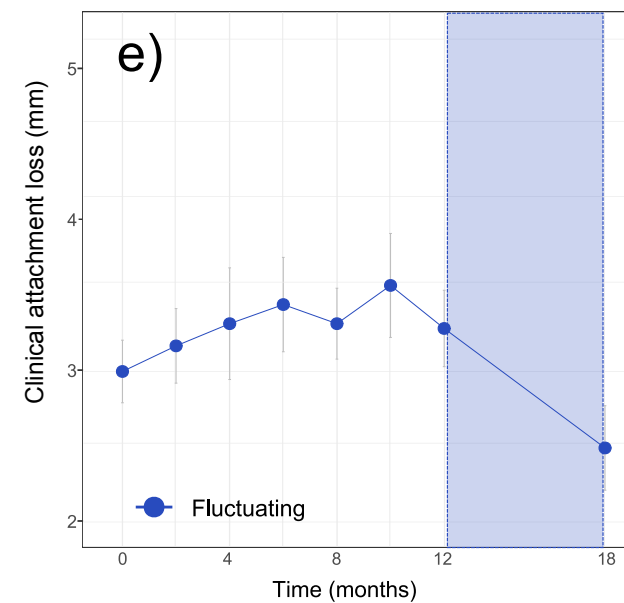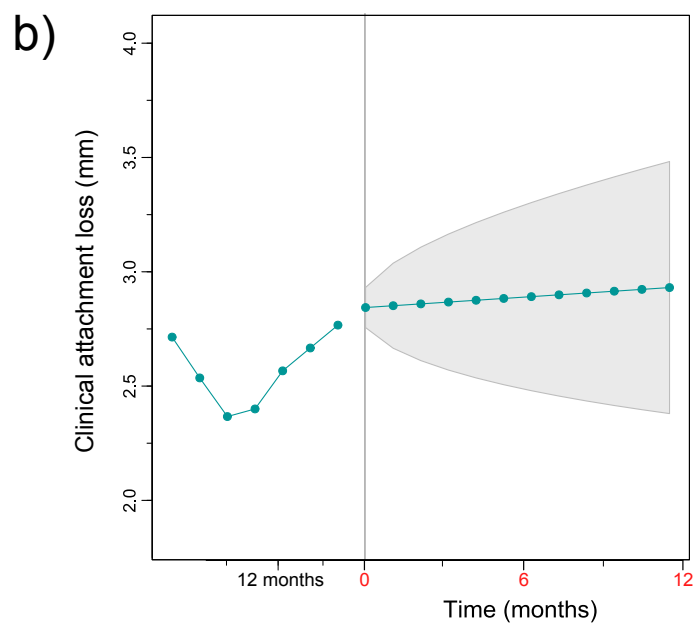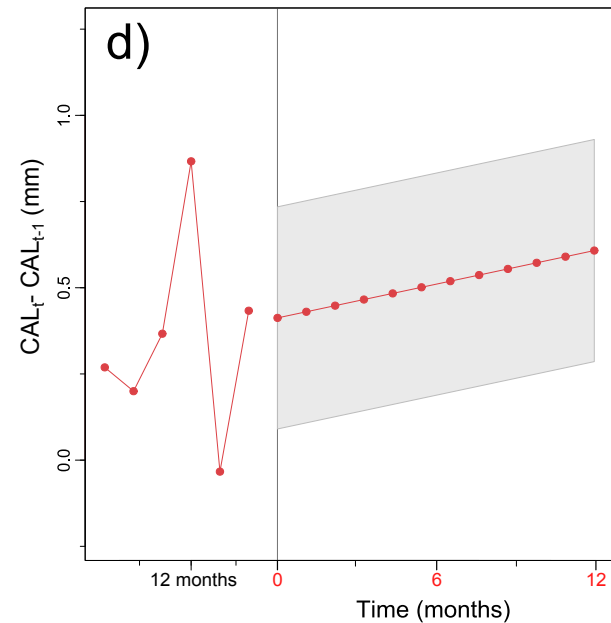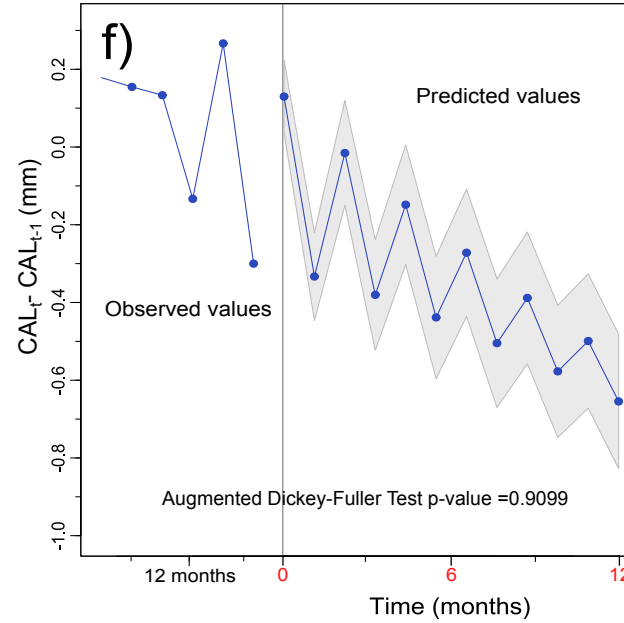

Supplement: Supplementary file 2 — Additional File 2: Figure S2. Time-series forecasting of clinical attachment loss (CAL) based on the observed results. a) Observed results of CAL in the stable samples before treatment. b) ARIMA forecast results for the stable sites. c) Observed results of CAL in progressing samples before treatment. d) ARIMA forecast results for the progressing sites. e) Observed results of CAL in fluctuating samples before treatment. f) ARIMA forecast results for the fluctuating sites. Grey zones represent 95% confidence intervals. Blue zones represent the period after periodontal treatment. [file 12915_2021_1169_MOESM2_ESM.pdf]

a)

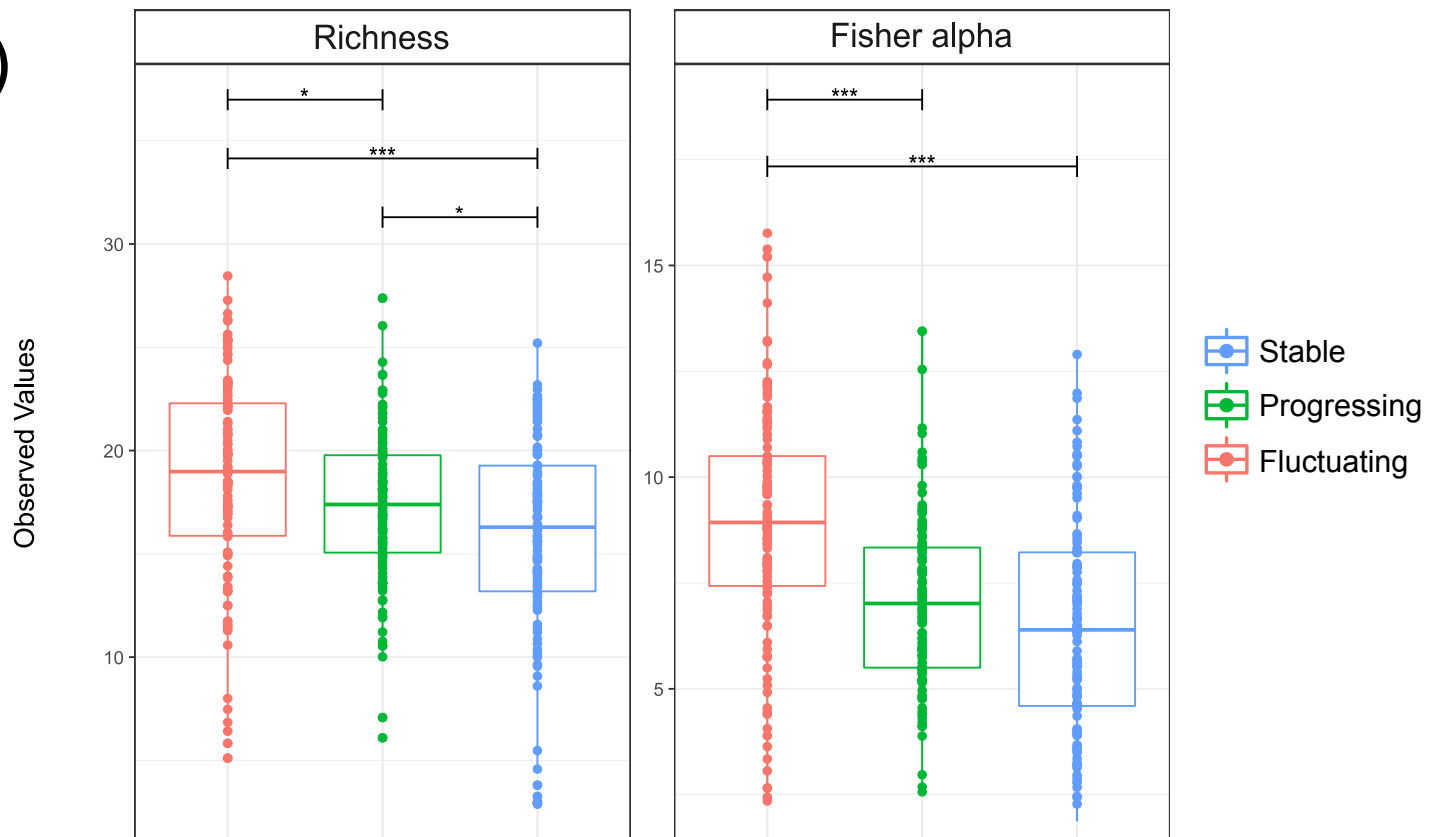

b)

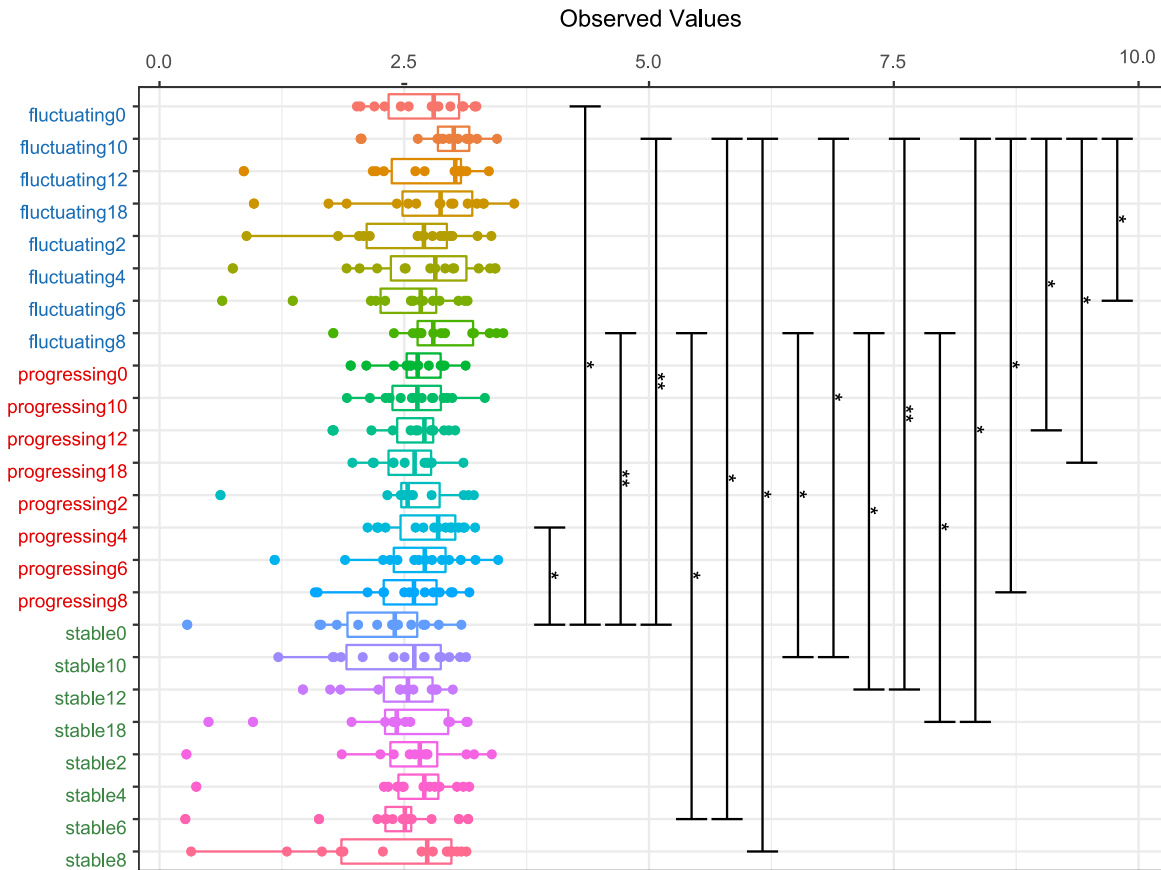

Supplement: Supplementary file 3 — Additional File 3: Figure S3. Αlpha-diversity results. a) α-diversity of all samples in the three different groups measured as richness and Fisher index. b) ANOVA results for Shannon diversity comparisons. Levels of significance (p < 0.05, p < 0.01, p < 0.001) were marked by one, two and three asterisks, respectively. [file 12915_2021_1169_MOESM3_ESM.pdf]

a)

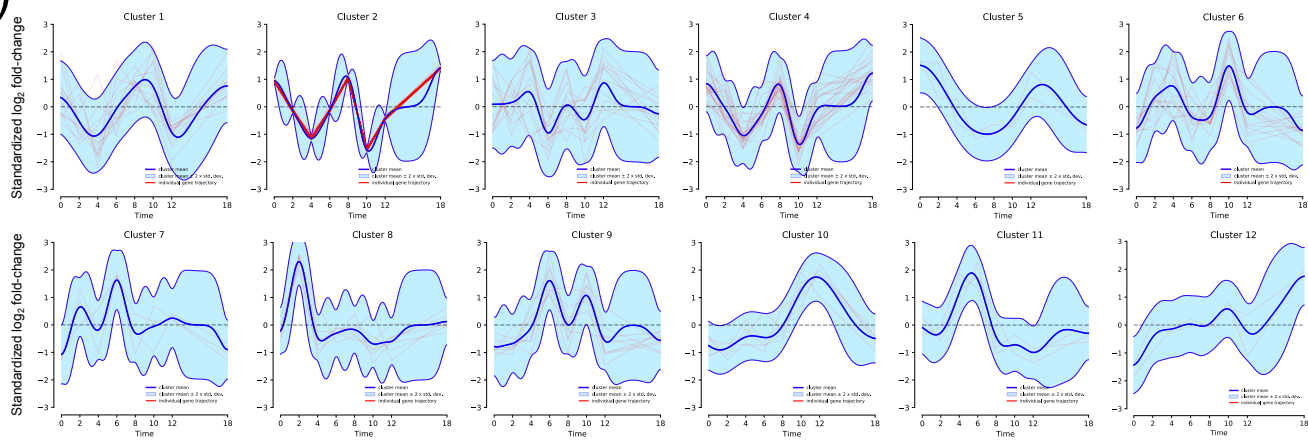

b)

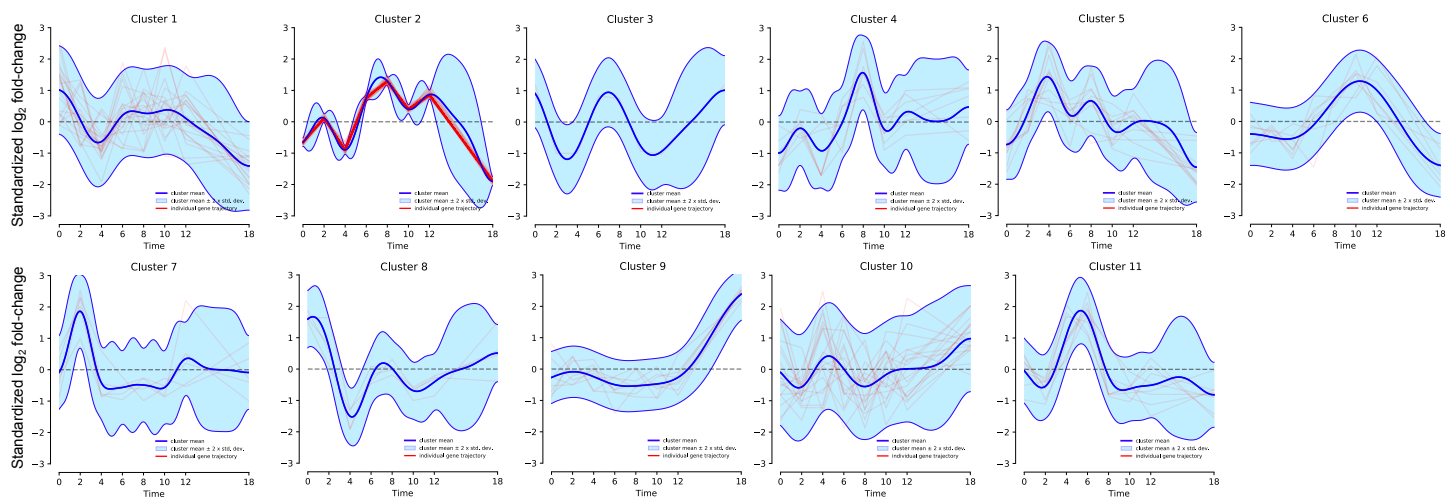

c)

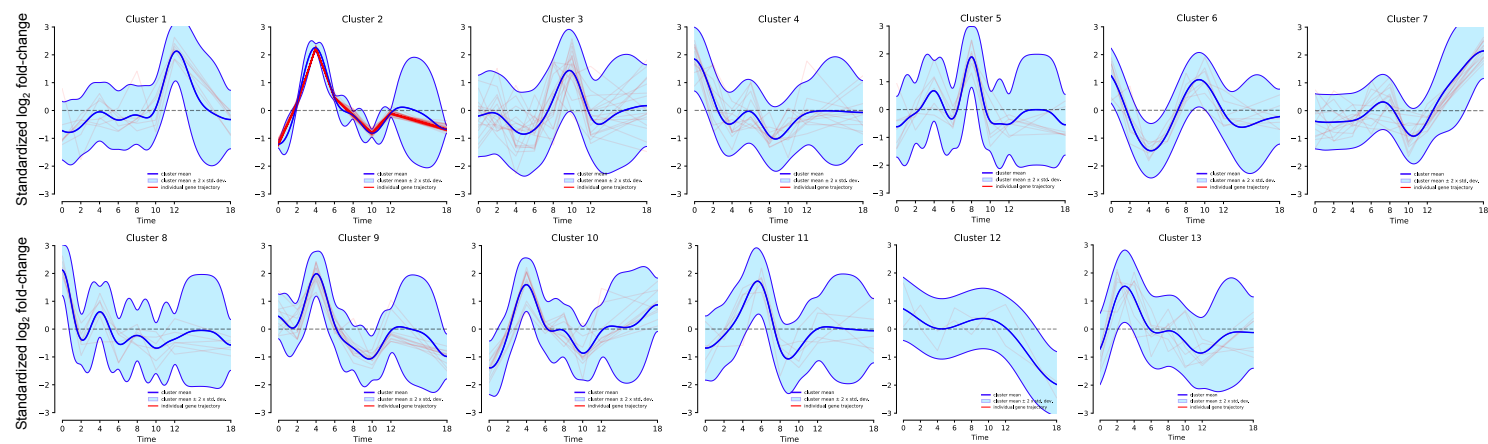

d)

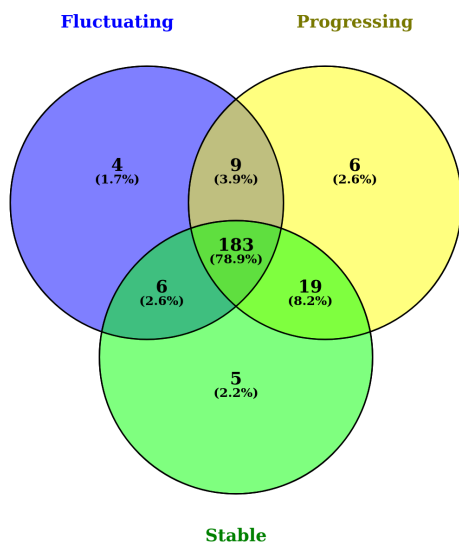

e)

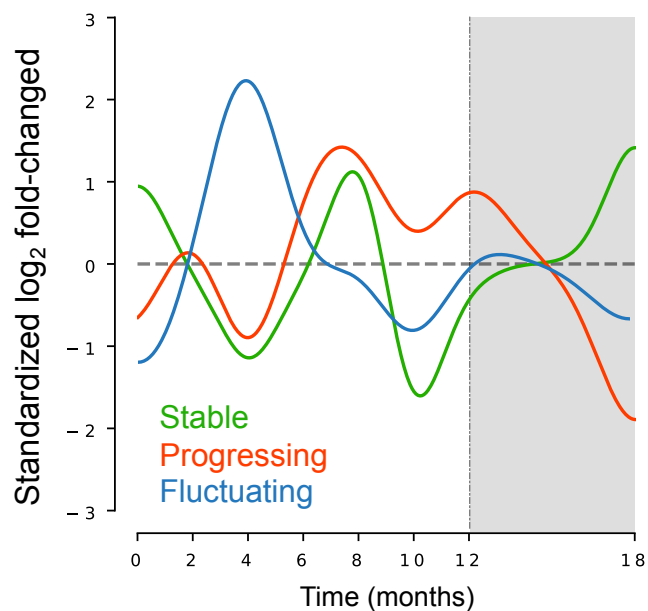

Supplement: Supplementary file 6 — Additional File 6: Figure S5. Dirichlet process Gaussian process mixture model (DPGP) clusters in species abundance trajectories. a) Clusters corresponding to stable samples. b) Clusters corresponding to progressing samples. c) Clusters corresponding to fluctuating samples. d) Venn-diagram of cluster 2, the largest cluster, shows a large group of organisms shared by the three groups from the three different groups. e) Trajectory of cluster 2 in the three groups. [file 12915_2021_1169_MOESM6_ESM.pdf]
