# Supplementary material for: Long-term dynamics of the human oral microbiome during clinical disease progression
Source: BMC Biol. 2021 Nov 6;19:240. doi: 10.1186/s12915-021-01169-z (PMC8572441; doi:10.1186/s12915-021-01169-z)
Supplement: Supplementary file 4 — Additional File 4: Figure S4. β-diversity of microbial communities. Multidimensional clustering was performed using PCoA with three dissimilarity distances: a) Bray-Curtis, b) Jaccard, and c) weighted-Unifrac. β-dispersion was calculated by computing the average distance of individual groups to the group centroid. Permutation analysis of variance (PERMANOVA) and corresponding r-squared and p-values are calculated on the β-dispersion between all possible pairwise combinations of the grouping variable levels. [file 12915_2021_1169_MOESM4_ESM.pdf]

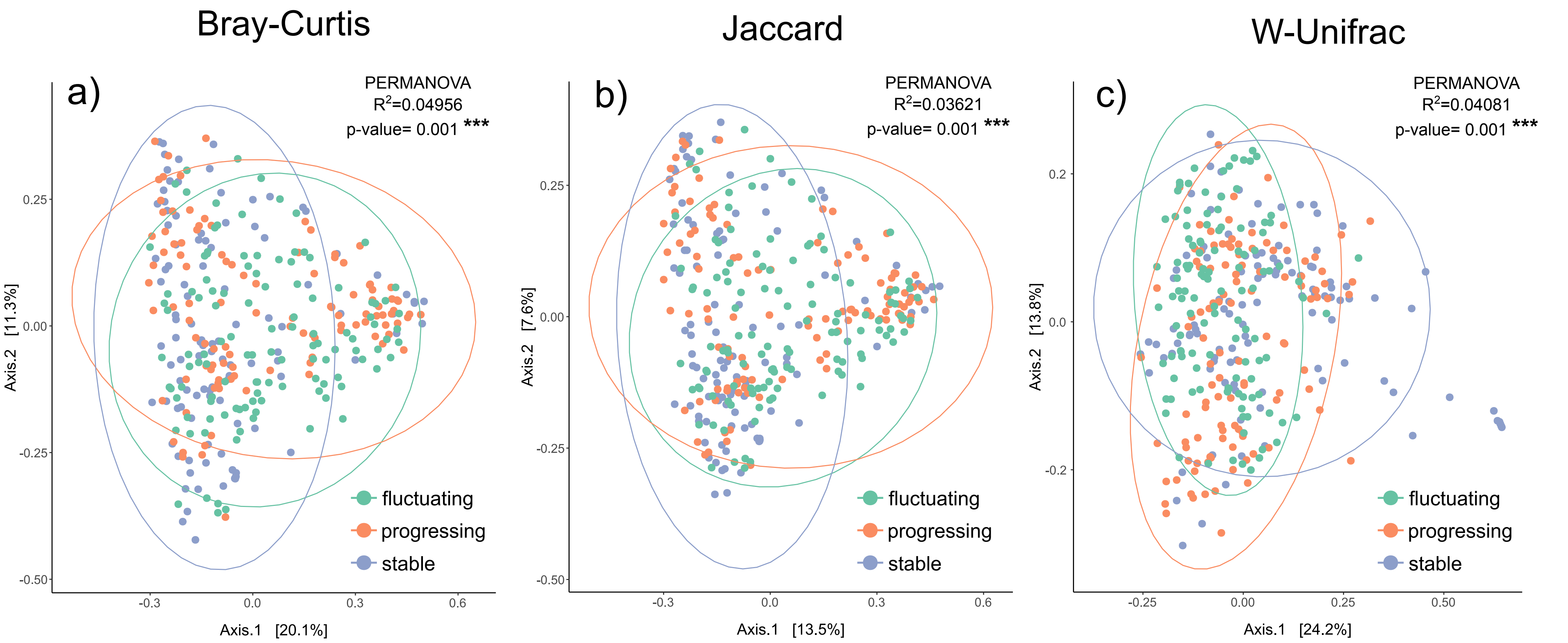

BETA-DISPERSION
(Observed p-value below diagonal, permuted p-value above diagonal)

	fluctuating	progressing	stable
fluctuating		0.792000	**0.035***
progressing	0.819989		**0.045***
stable	**0.029624***	**0.045075***	

BETA-DISPERSION
(Observed p-value below diagonal, permuted p-value above diagonal)

	fluctuating	progressing	stable
fluctuating		0.959000	**0.048***
progressing	0.966042		**0.041***
stable	**0.039338***	**0.041805***	

BETA-DISPERSION
(Observed p-value below diagonal, permuted p-value above diagonal)

	fluctuating	progressing	stable
fluctuating		0.126000	0.393
progressing	0.136337		**0.020***
stable	0.400459	**0.019993***	

Signif. codes: *** 0.001 ** 0.01, * 0.05, '.' 0.1
